# Object/Context Specific Memory Deficits following Medial Frontal Cortex Damage in Mice

**DOI:** 10.1371/journal.pone.0043698

**Published:** 2012-08-21

**Authors:** Simon C. Spanswick, Richard H. Dyck

**Affiliations:** 1 Department of Psychology, University of Calgary, Calgary, Alberta, Canada; 2 Hotchkiss Brain Institute, University of Calgary, Calgary, Alberta, Canada; University of Lethbridge, Canada

## Abstract

Recent evidence suggests that the medial prefrontal cortex (MFC) is important for processing contextual information. Here we evaluate the performance of mice with MFC damage in a discrimination task that requires an association between an object and the context in which it was experienced (the object/context mismatch task), as well as a version of the novel object preference task that does not require knowledge of contextual information to resolve. Adult C57/BL6 mice received aspiration lesions of the MFC or control surgery. Upon recovery, mice were tested in the object/context mismatch and novel object preference tasks. The object/context mismatch task involved exposing mice to two different contexts, each of which housed a unique pair of identical objects. After a brief delay, mice were re-exposed to one of the contexts, this time with one object that was congruent with that context and one that was not. Novel object preference was performed within a single context, housing an identical pair of objects. After the initial exposure and following a brief delay, mice were re-exposed to the context, this time housing a familiar and a novel object. Control mice were able to successfully resolve the object/context mismatch and novel object preference discriminations, investigating the incongruent/novel object within each task significantly greater than chance. Mice with MFC damage experienced deficits in the object/context mismatch task but not the novel object preference task. These findings add to a growing body of evidence that demonstrate a critical role for the MFC in contextual information processing.

## Introduction

Recent evidence has implicated the medial prefrontal cortex (MFC) as an important structure in processing episodic-like [1], [2], [3], contextual [4], [5], and relational memory [6] in rodents. Lesions targeting the MFC are sufficient to disrupt the ability of rats and mice to successfully perform a number of behaviours, including a “what-where-when” version of an object recognition task [2], contextual fear memory [5], and its extinction [4].

Devito et al. [6] recently assessed the effects of MFC lesions in mice on an odor-based version of the transitive inference task. Briefly, mice were trained to discriminate between several pairs of overlapping odor cues, one of which was paired with reward (for example A+ vs. B−, B+ vs. C−, C+ vs. D−, D+ vs. E−). When the odor discriminations had been successful learned, mice were presented with a transitive inference probe (two indirectly related odors, for example B vs. D). Devito et al. [6] report significant odor discrimination impairments in mice with ibotenic acid lesions limited to the MFC, only when successful discrimination relied upon intact transitive inference. Deficits in transitive inference have typically been associated with damage to the hippocampus [7], [8], [9], and entorhinal cortex [10] in non-human animals. The results of the Devito et al. [6] study, combined with the findings from studies assessing the effect of hippocampus damage in the same task [7], [8], [9], led them to propose that a dynamic interplay between the MFC and hippocampus is necessary to support relational memories.

The findings from MFC lesion studies are mirrored by experiments utilizing immunohistochemical [1], [7] and electrophysiological [4] techniques to measure neuronal activity in response to contextually relevant information. Using c-Fos expression as an indicator of cellular activity, Knapska and Maren [11] show contextually dependent activation of MFC neurons (in addition to expression in amygdala and hippocampus) during extinction of conditioned fear memory in rats. They suggest that the MFC, in conjunction with amygdalar and hippocampal circuitry, is important for mediating the contextual specificity of the extinction process. Taken together, the available data suggest that the MFC is part of a system responsible for encoding at least some of the components of episodic-like memory in the rodent [3], [6].

Many of the tasks used to assess MFC function involve relatively complicated behavioral designs, utilizing modifications to a contextual fear paradigm [5], discrimination of multiple odor pairs [6], or visual objects [2]. Here we employ a simple method, using two different versions of a spontaneous exploration task (novel object preference and object/context mismatch) to assess the effects of lesions focused on the MFC on the behavior of C57/BL6 mice. As spontaneous exploration tasks rely on the natural propensity of animals to investigate novel objects, they do not involve aversive or extended training protocols, and have the additional advantage of avoiding food or water restriction. Specifically, we employ a task that requires an association between an object and the context in which it was experienced (object/context mismatch task), as well as a version of a novel object preference task that does not require contextual information to resolve. The object/context mismatch task has previously been employed to assess contextually dependent discrimination behaviour in rats with damage limited to the hippocampus [12], [13]. Rats with hippocampal damage are impaired in the object/context mismatch task, but are able to successfully resolve a more standard version of a novel object preference task, as long as contextual information is not required for the discrimination [12], [13], [14], [15].

Prior research has demonstrated that damage limited to the MFC does not impair performance in a standard version of novel object preference in rodents [16], [17]. In the object-in-place task, in which an animal is required to detect an object relative to surrounding objects as well as its location, MFC damage results in discrimination impairments in rodents [16], [18], as well as non-human primates [19]. Given the recent findings regarding the role of the rodent MFC in contextual memory [4], [5], and the strong anatomical connections between the rodent hippocampus and MFC [20], [21], we sought to assess the ability of C57/BL6 mice with aspiration lesions of the MFC (prelimbic, infralimbic, and anterior cingulate cortices, [22]) to resolve the object/context mismatch task. Our goal was to demonstrate with a simple behavioural assay that the MFC is important for contextual information processing in the mouse.

## Results

**Figure 1:**
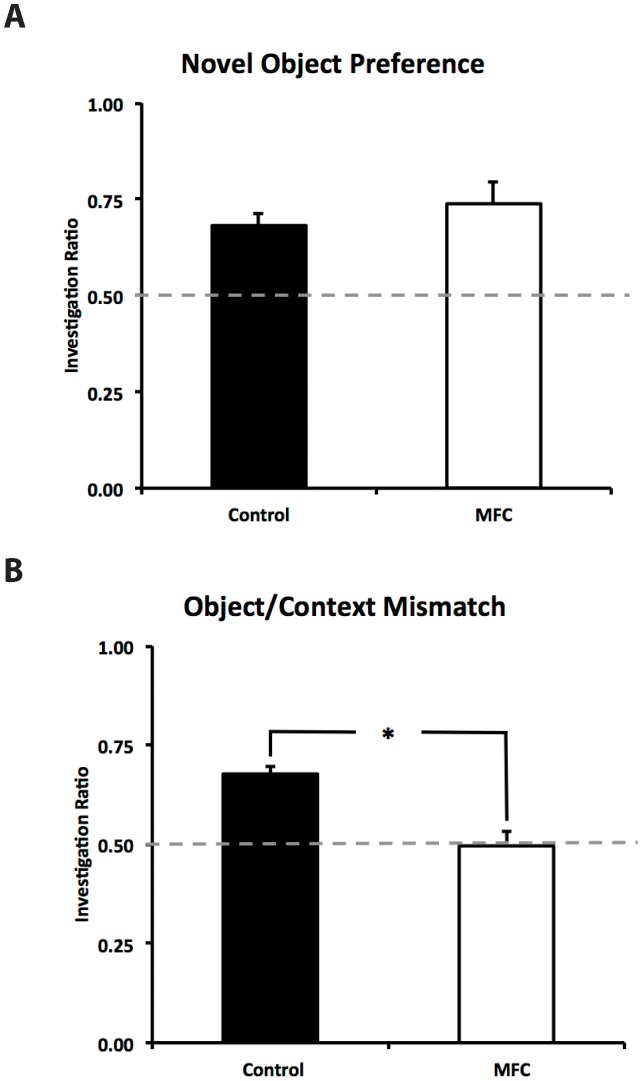
Performance of mice in two versions of a spontaneous exploration task. Investigation ratios were calculated by dividing the amount of time spent investigating the novel object by overall object investigation time. An investigation ratio of 0.50 represents chance. (A) Control and MFC-damaged mice investigated the novel object at significantly greater than chance levels in the novel object preference task. The investigation ratios of the two groups did not differ significantly from one another. (B) Control mice were able to successfully perform the object/context mismatch task, investigating the novel object/context pairing significantly more than chance. Mice with MFC damage were significantly impaired relative to controls and did not investigate the novel object/context pairing significantly more than chance. Asterisk denotes significance, p = 0.004.

Eleven mice were used in the final statistical analysis as 2 control mice were removed from the study due to lack of object investigation (more than 3 standard deviations below the mean), this resulted in a control group consisting of 5 mice and a group of 6 MFC damaged mice. An alpha level of 0.05 was used for all statistical analyses, with all means reported as plus/minus standard error of the mean. Control and MFC mice did not differ significantly in their investigation of the identical pair of objects during the learning phase of the novel object preference task, F(1,9) = 0.872, p = 0.375. Control mice spent 24.54±5.84 seconds investigating the identical objects, with MFC mice investigating the objects for 21.96±4.78 seconds. To determine if investigation ratios differed from chance (0.50) for each group, single sample t tests were run. During the test phase, control mice investigated the novel object at significantly greater than chance levels, t(4) = 5.053, p = 0.007, d = 3.00, as did MFC mice, t(5) = 3.879, p = 0.012, d = 1.64. Control and MFC mice had mean investigation ratios of 0.68±0.03 and 0.74±0.06 respectively. These investigation ratios did not differ significantly from one another, F(1,9) = 0.302, p = 0.596 (Figure 1A).

Control and MFC mice did not differ significantly in their object investigation during learning phase one (F(1,9) = 0.091, p = 0.77), or learning phase two of the object/context mismatch task (F(1,9) = 0.094, p = 0.766). Control mice spent a mean of 27.64±3.58 seconds investigating the object pair during learning phase one, with MFC mice investigating for 30.30±7.29 seconds. Control and MFC mice spent 32.24±4.52 seconds and 29.62±6.62 seconds investigating the objects during learning phase two, respectively. During the test phase, control mice investigated the novel object/context pairing significantly greater than chance, t(4) = 8.429, p = 0.001, d = 3.60 with an investigation ratio of 0.68±0.02. Mice with MFC lesions did not investigate the novel object/context pairing significantly different from chance, t(5) = −0.131, p = 0.901 (investigation ratio of 0.50±0.04). The mean investigation ratio during the object/context mismatch task differed significantly between control and MFC damaged mice, F(1,9) = 15.267, p = 0.004, d = 1.53 (Figure 1B). Statistical analysis of overall investigation time during the test phase of object/context mismatch revealed no significant difference between controls and MFC damaged mice, F(1,9) = 0.111, p = 0.746.

**Figure 2:**
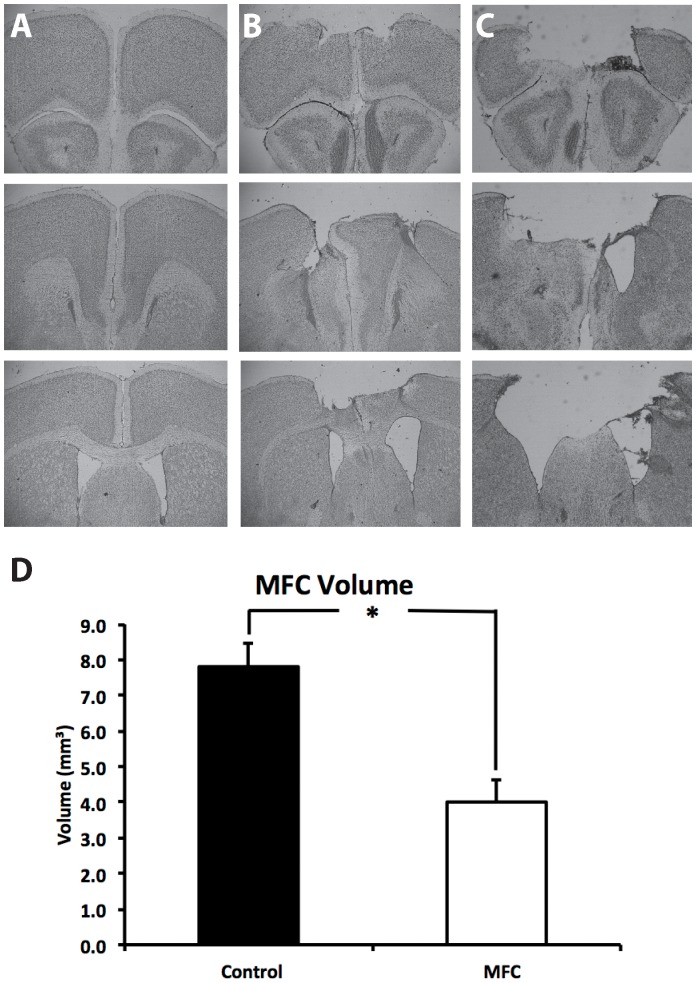
Representative Cresyl Violet stained sections from a control mouse (A), a mouse with a small MFC lesion (B), and a large MFC lesion (C). Images taken at 2.5X, approximately 2.68 mm, 1.70 mm, and 0.50 mm anterior to Bregma. (D) Cavalieri volume estimates show that aspiration lesions resulted in approximately 50% loss of the MFC. Asterisk denotes significance, p = 0.002.

Cavalieri volume analysis revealed that control mice had a mean MFC volume of 7.79±0.74 mm^3^, whereas those mice that received aspiration lesions of the MFC had a significantly smaller volume (F(1,9) = 20.093, p<0.01, d = 1.59, 4.01±0.67 mm^3^), representing an average loss of approximately 50% of the MFC (photomicrographs of representative lesions included in Figure 2). MFC damage varied from a low of 21%, to a high of 77% loss of MFC volume relative to controls. Smaller lesions typically damaged the anterior cingulate cortex, sparing some of the prelimbic cortex, and the majority of the infralimbic cortex below. Large lesions of MFC removed all of the cingulate cortex, the prelimbic cortex, and most of the infralimbic cortex. Lesion size was not significantly correlated with performance in the object/context mismatch task, *r*(6) = −.283, p = 0.586.

## Discussion

Control mice were able to successfully resolve both the novel object preference and object/context mismatch tasks, investigating the novel object and the novel object/context pairing at significantly greater than chance levels. In this experiment control mice investigated the novel object in the object preference task at levels similar to other reports of investigation behaviour within the mouse [23]. Historically the object/context mismatch task has been employed to assess discrimination behaviour in rats [12], [13], [14], [15]. Here we show that intact C57/BL6 mice are able to perform the object/context task, exhibiting an investigation ratio similar to prior reports [12], [13]. As such, the object/context mismatch may provide a quick and sensitive measure of contextually dependent discrimination ability in any number of mouse models.

Two of seven control mice were removed from the experiment due to inadequate investigation time. This was based on our a priori exclusion criteria of three standard deviations below the mean investigation time. Our method differs somewhat from other published criteria, which typically employ a cutoff, ranging from one to five seconds of total investigation time for both the target and sample objects [14], [24]. Both of the mice in question investigated the objects for less than three seconds, falling within the range of cutoff scores mentioned above. Our dropout rate is higher than what is typically reported for the object/context mismatch task in rats [12], [13], suggesting that this may be a species-specific issue. Interestingly, mice and rats seem to perform similarly in regards to the novel object preference task [25], suggesting that this may be a result of the unique demands of the object/context mismatch task. Others have shown that increasing “memory load” within a standard version of the novel object preference task decreases the ability of mice to successfully discriminate between a novel and previously encountered object [26]. As such, the added complexity of multiple contexts and object/context associations may account for the increase in dropout within our control group.

Mice with lesions of the MFC discriminated between a previously encountered and a novel object, doing so at levels similar to intact controls. Successful performance of the novel object preference task by MFC damaged mice is not surprising given the extensive literature demonstrating that novel object preference is dependent upon intact hippocampal or perirhinal cortex circuitry [27], [28], [29]. This is corroborated by studies using lesion [17], [30] or disconnection [16] methodologies showing that MFC disruption does not impair simple novel object preference. This anterograde assessment of MFC damage on novel object preference, in combination with data showing that damage to the hippocampus or perirhinal cortices produces discrimination impairments, is consilient with the idea that the MFC may not be involved in novel object preference. However, to fully rule out the role of the MFC in novel object preference, a retrograde examination is required [31], as other structures may be able to support successful novel object preference in the absence of the MFC.

We utilize a relatively short time interval (5 min) between the learning and test phase of the novel object preference task. This was done to equate the period spent in the transport tub in the novel object preference task and the object/context mismatch task, the latter of which requires a brief epoch between the learning and test phases. Despite this, our results are similar to those that employ longer intervals in the novel object preference task [23], suggesting that changing the elapsed time between the learning and test phases does not alter the ability of MFC damaged mice to discriminate between previously encountered and novel objects.

Only when the discrimination required knowledge of context did MFC damaged mice falter, failing to investigate the novel object/context pairing at greater than chance levels. Lack of overall object investigation does not account for this failure, given the fact that during the learning and test phases of the object/context mismatch task MFC damaged mice investigated the objects as much as controls. Our findings are congruent with other reports of MFC damage-induced contextual impairments in mice [2], and rats [4], [5] in other tasks.

Barker et al. [16] report that MFC lesions do not disrupt the performance of rats in a standard version of the novel object preference task. Yet when manipulations are made to surrounding proximal objects (local context) in the object-in-place task, rats with MFC damage fail to discriminate at levels significantly greater than chance. The results we present here are similar; mice with MFC damage were able to successfully discriminate between a novel and previously encountered object, only when contextual information was not required. However, we are the first to demonstrate that MFC damage results in discrimination impairments specifically in the object/context mismatch task.

Studies assessing the discrimination ability of rats with lesions of the hippocampus [12] or dentate gyrus [13] in the object/context mismatch task have shown that these structures are critical for its resolution. Here we demonstrate that in mice, MFC damage is sufficient to induce deficits within the same task. Although we did not directly test the effect of hippocampal lesions on mice in the object/context mismatch task, our findings are congruent with a body of literature showing that the MFC and hippocampus contribute to relational memory that includes contextual information [2]. Devito et al. [6] suggest that the hippocampus is responsible for encoding and retrieval of elemental information and the context in which they were experienced. Furthermore, they suggest that the MFC contributes to relational memory by monitoring the match between the retrieved items and contextual information, providing a mechanism by which detection of novelty may occur. Disruption of either structure is therefore likely to result in impairments in contextually dependent tasks.

It is important to note that in some cases aspiration of the MFC resulted in damage to the adjacent motor cortices, potentially altering motor function in MFC damaged mice. Evidence from tract tracing studies in rats show that projections arising in the MFC terminate in several motor areas [32]. Altered motor function as a result of incidental damage to the motor cortex, or disruption of the MFC itself, may therefore alter the investigation capabilities of MFC-damaged mice. It is likely that damage to the surrounding motor cortices would result in generalized deficits in motor behaviour. Mice with MFC damage investigated the objects as much as controls in both the learning and test phases in each of the behavioural tasks we employed. Only during the test phase of the object/context mismatch task did the *ratio* of investigation differ between control and MFC damaged mice. This suggests that investigation behaviour in general was unaffected by incidental motor cortex damage, as only when knowledge of context was required did the behaviour of MFC damaged mice falter. The lack of a significant correlation between lesion size and performance in the object/context mismatch task suggests that even when surrounding motor cortices are left intact, behaviour is impaired. This result is bolstered by other studies, that also do not report a significant correlation between MFC lesion size and behavioural deficit [2]. Our findings fit with a growing body of evidence, employing multiple lesion techniques, showing that the MFC is important for contextual information processing [4], [5].

Interestingly, the effects of MFC disruption are not solely limited to tasks requiring knowledge of contextual information. Devito and Eichenbaum [3] recently showed that mice with ibotenic acid lesions of the MFC are impaired on an odor-based temporal order task. Similar results have been obtained in MFC-damaged rats [30], [33]. For example, using a temporary lesion strategy (infusion of CNQX, scopolamine, or AP5), Barker et al. [33] show that MFC disruption is sufficient to impair temporal order memory performance for visual stimuli. We attempted to control for order effects by counterbalancing the MFC and control groups within our experiment. As the number of mice in the control group was uneven, a completely counterbalanced group was not possible. To determine if temporal order was responsible for discrimination ability within our controls, we assessed the effects of the order of testing, and found no differences between groups (data not shown). This, combined with our counterbalancing within the MFC group, is evidence that our mice are unlikely to be resolving the discrimination using temporal cues.

The ability of mice with MFC damage to perform the novel object preference task suggests that the MFC may not be necessary for the detection of novelty *per se*. Instead, the MFC is critical in situations in which a novel association of objects and contexts is required. Studies assessing other modalities of relational memory indicate that the role of the MFC extends beyond the realm of context [6]. The convergent data from this, and similar studies, suggests that the MFC is part of a system responsible for encoding at least some of the components of episodic-like memory rodents [3], [6]. The present study provides a new, simple method by which to analyze the behavioural consequences of MFC damage in the mouse. Furthermore, it adds to a growing body of evidence demonstrating that the MFC plays a critical role in relational or episodic-like memories.

## Materials and Methods

All of the experimental procedures were approved under Protocol #BIO8R-02 by the University of Calgary Animal Care Committee, and were performed in accordance with the Canadian Council on Animal Care guidelines. Thirteen male C57/BL6 mice were obtained from the University of Calgary breeding colony. At the onset of the experiment all mice were 60 days of age. Mice were housed in groups (3–5 per cage) in a 12-hour light/dark cycle and had *ad libitum* access to food and water through the duration of the experiment.

Mice received an aspiration lesion of the medial frontal cortex or corresponding control surgery. Anesthesia was induced via an intramuscular injection of ketamine/xylazine (50 mg/kg). When a stable anesthetic plane had been achieved the mouse was secured in a stereotaxic apparatus (Kopf®). A section of skull between Bregma and the nasofrontal suture extending approximately 2 mm laterally from midline was trephined, exposing the surface of the brain. Using controlled vacuum, the exposed area of brain was removed by aspiration, to a depth of approximately 1.5 mm, until the white matter of the corpus callosum was visible at the posterior-most region. When bleeding had ceased, the wound was sutured and the mouse was allowed to recover in its home-cage, which was placed on a heating pad maintained at 37°C. Mice were housed in isolation post-surgically and allowed to recover for 7 days, after which they were returned to group housing where they remained for a further 7 days prior to initiating behavioral testing.

All mice were tested in a version of the novel object preference task and the object/context mismatch task [12]. Exposure to the tasks was counterbalanced to minimize order effects. Mice were tested in a similar manner as has been described before [13]. The contexts consisted of 2 white square plastic boxes (40 cm×40 cm×40 cm), one of which had a circular, black plastic insert (approximately 40 cm in diameter), both of the contexts had standard housing bedding on the floor. The contexts were housed in separate rooms, each of which had a unique combination of distal cues on the walls. Context A (white, square box) was housed in a brightly lit testing room, whereas context B (black, circular container) was housed in a dimly lit room.

**Figure 3:**
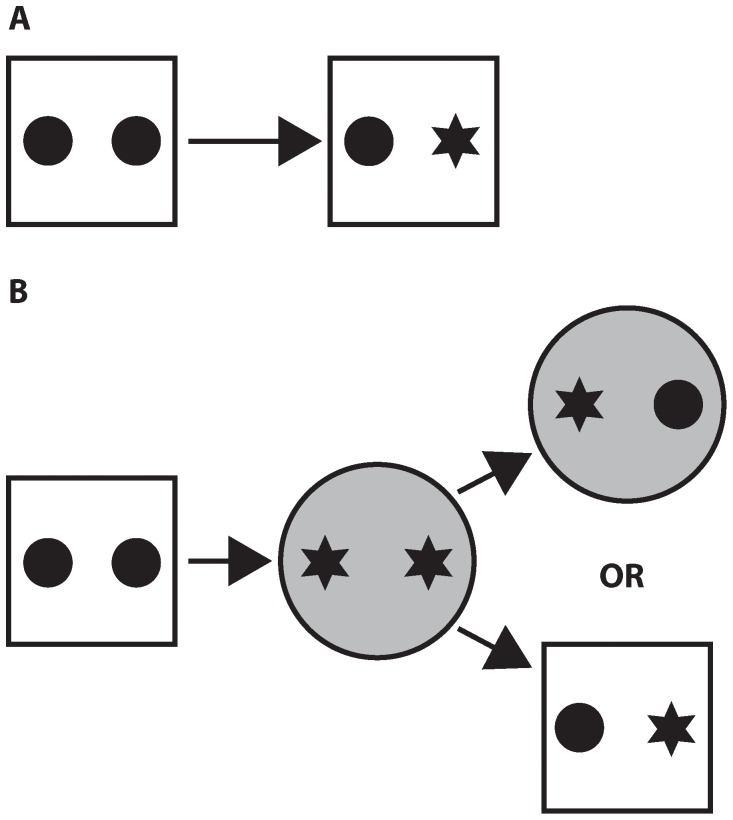
Schematics of the spontaneous exploration tasks. (A) Novel object preference. Mice were allowed to investigate two identical objects for 5 minutes. After a 5-minute delay, mice were returned to the same context. This time the context housed an identical copy of one of the previously encountered objects, in addition to a novel object. Each mouse was allowed to explore for 3 minutes, during which they were videotaped. (B) The object/context mismatch task. Mice were exposed to two different contexts, one immediately after the other, each of which housed a unique pair of objects. After a 5-minute delay, mice were returned to one of the contexts, this time containing a congruent and incongruent object. As with the novel object preference task, mice were given 3 minutes to explore.

Context A was employed for all novel object preference sessions. Mice were exposed to the context, devoid of any objects, for 2 days (10 min each day) prior to testing. Approximately 5 minutes prior to each exposure, the walls of the context were wiped down with a 70% ethanol solution. On test day (day 3), 2 identical copies of an object were placed in the context. Mice were allowed 5 min to explore the pair of objects (learning phase) and then returned to a transport cage for a period of 5 min. A new, yet identical copy of one of the previously encountered objects, along with a novel object, were placed in the context (Figure 3A). Mice were returned to the context and allowed to explore for 3 min (test phase). All exploration sessions were recorded with a video camera (Sony Digital Handycam) mounted on a tripod. Video sessions were visually inspected, employing a stopwatch to quantify object investigation. Object investigation was operationalized as the mouse placing its nose within approximately two centimeters of the object, while facing it. Standing on an object was not scored as investigation. An investigation ratio was calculated by dividing the time spent investigating the novel object by the time spent investigating both objects. As such, an investigation ratio of 0.50 represented chance levels, or no preference.

For the object/context mismatch task, mice were exposed to context A and context B, one immediately after the other for 10 min a day, for 2 days immediately prior to testing. On test day (day 3) each context contained a unique pair of identical objects. Mice explored each of the contexts for 5 minutes, one immediately after the other (learning phase 1 and 2). After exposure to both contexts mice were returned to their transport cage for 5 minutes. After the 5-minute delay mice were returned to one of the contexts, this time with one object from each, and allowed to explore for 3 minutes (test phase, Figure 3B). Exposure to contexts, object location, and object/context association were counter-balanced. As mentioned above, all exploration sessions were video recorded and investigation ratios were calculated off-line for all mice.

Upon conclusion of behavioral testing the mice were injected intraperitoneally with an overdose of sodium pentobarbital (400 mg/kg) and perfused transcardially with 0.1 M phosphate buffered saline (PBS, 15 ml), followed by a solution of 4% paraformaldehyde in PBS (25 ml). Brains were extracted and stored in the same 4% paraformaldehyde solution at 4°C for 24 hours. The brains were then transferred to a 30% sucrose/PBS solution until ready to cut with a freezing sliding microtome (American Optical, model #860; Buffalo, NY, USA). Forty-micron thick sections were collected throughout the rostral/caudal extent of the medial frontal cortex, employing a section-sampling fraction of 1/6.

A single series of tissue from each mouse was slide mounted and stained with Cresyl Violet, yielding approximately 15 sections throughout the extent of the medial frontal cortex. The volume of the medial frontal cortex was calculated in control and aspiration lesion mice employing the Cavalieri method [34], [35]. Images of brain sections were captured using a Zeiss Axioskop 2 microscope attached to a Qimaging camera (QICAM 10-bit) using a 2.5×/0.075 objective. ImageJ software (http://rsb.info.nih.gov/ij/) was utilized to position a random, systematic sampling grid over each image. An area per point of 0.08 mm^2^ was determined as sufficient, yielding approximately 400 contact points between the grid and medial frontal cortex in control mice. The total number of contact points between the medial frontal cortex and the grid was quantified for each section. The number of contact points per section was multiplied by the area associated with each point (0.08 mm^2^), the section cut thickness (40 microns), and the section sampling fraction (1/6). These numbers were then summed to provide an estimated total volume of the MFC.

## References

[1] Bossert JM, Stern AL, Theberge FR, Cifani C, Koya E, et al (2011) Ventral medial prefrontal cortex neuronal ensembles mediate context-induced relapse to heroin. Nat Neurosci 14: 420–2. 21336273 10.1038/nn.2758 PMC3077927

[2] Devito LM, Eichenbaum H (2010) Distinct contributions of the hippocampus and medial prefrontal cortex to the “what-where-when” components of episodic-like memory in mice. Behav Brain Res 215: 318–25. 19766146 10.1016/j.bbr.2009.09.014 PMC2891645

[3] Devito LM, Eichenbaum H (2011) Memory for the order of events in specific sequences: contributions of the hippocampus and medial prefrontal cortex. J Neurosci 31: 3169–75. 21368028 10.1523/JNEUROSCI.4202-10.2011 PMC3081724

[4] Judo C, Matsumoto M, Yamazaki D, Hiraide S, Yanagawa Y, et al (2010) Early stress exposure impairs synaptic potentiation in the rat medial prefrontal cortex underlying contextual fear extinction. Neuroscience 169: 1705–14. 20600655 10.1016/j.neuroscience.2010.06.035

[5] Li JS, Hsiao KY, Chen WM (2011) Effects of medial prefrontal cortex lesions in rats on the what-where-when memory of a fear conditioning event. Behav Brain Res 218: 94–8. 21115067 10.1016/j.bbr.2010.11.044

[6] Devito LM, Lykken C, Kanter BR, Eichenbaum H (2010) Prefrontal cortex: role in acquisition of overlapping associations and transitive inference. Learn Mem 17: 161–7. 20189961 10.1101/lm.1685710 PMC2832922

[7] Dusek JA, Eichenbaum H (1997) The hippocampus and memory for orderly stimulus relations. Proc Natl Acad Sci 94: 7109–7114. 9192700 10.1073/pnas.94.13.7109 PMC21293

[8] Van der Jeugd A, Goddyn H, Laeremans A, Arckens L, D’Hooge R, et al (2009) Hippocampal involvement in the acquisition of relational associations, but not in the expression of a transitive inference task in mice. Behav Neurosci 123: 109–114. 19170435 10.1037/a0013990

[9] Devito LM, Kanter BR, Eichenbaum H (2010) The hippocampus contributes to memory expression during transitive inference in mice. Behav Brain Res 20: 108–117. 10.1002/hipo.20610 PMC2801762 19405137

[10] Buckmaster CA, Eichenbaum H, Amaral DG, Suzuki WA, Rapp PR (2004) Entorhinal cortex lesions disrupt the relational organization of memory in monkeys. J Neurosci 44: 9811–9825. 10.1523/JNEUROSCI.1532-04.2004 PMC6730224 15525766

[11] Knapska E, Maren S (2009) Reciprocal patterns of c-Fos expression in the medial prefrontal cortex and amygdala after extinction and renewal of conditioned fear. Learn Mem 16: 486–93. 19633138 10.1101/lm.1463909 PMC2726014

[12] Mumby DG, Gaskin S, Glenn MJ, Schramek TE, Lehmann H (2002) Hippocampal damage and exploratory preferences in rats: memory for objects, places, and contexts. Learn Mem 9: 49–57. 11992015 10.1101/lm.41302 PMC155935

[13] Spanswick SC, Sutherland RJ (2010) Object/context-specific memory deficits associated with loss of hippocampal granule cells after adrenalectomy in rats. Learn Mem 17: 241–5. 20410060 10.1101/lm.1746710 PMC2893217

[14] O’Brien N, Lehmann H, Lecluse V, Mumby DG (2006) Enhanced context-dependency of object recognition in rats with hippocampal lesions. Behav Brain Res 170: 156–62. 16580742 10.1016/j.bbr.2006.02.008

[15] Piterkin P, Cole E, Cossette MP, Gaskin S, Mumby DG (2008) A limited role for the hippocampus in the modulation of novel-object preference by contextual cues. Learn Mem 15: 785–791. 18832565 10.1101/lm.1035508

[16] Barker GR, Bird F, Alexander V, Warburton EC (2007) Recognition memory for objects, place, and temporal order: a disconnection analysis of the role of the medial prefrontal cortex and perirhinal cortex. J Neurosci 27: 2948–2957. 17360918 10.1523/JNEUROSCI.5289-06.2007 PMC6672574

[17] Mitchell JB, Laiacona J (1998) The medial frontal cortex and temporal memory: tests using spontaneous exploratory behaviour in the rat. Behav Brain Res 97: 107–113. 9867236 10.1016/s0166-4328(98)00032-1

[18] Kesner RP, Ragozzino ME (2003) The role of the prefrontal cortex in object-place learning: a test of the attribute specificity model. Behav Brain Res 146: 159–165. 14643468 10.1016/j.bbr.2003.09.024

[19] Browning PG, Easton A, Buckley MJ, Gaffan D (2005) The role of prefrontal cortex in object-in-place learning in monkeys. Eur J Neurosci 22: 3281–3291. 16367793 10.1111/j.1460-9568.2005.04477.x

[20] Jay TM, Witter MP (1991) Distribution of hippocampal CA1 and subicular efferents in the prefrontal cortex of the rat studied by means of anterograde transport of Phaseolus vulgaris-leucoagglutinin. J Comp Neurol 313: 574–86. 1783682 10.1002/cne.903130404

[21] Verwer RW, Meijer RJ, Van Uum HF, Witter MP (1997) Collateral projections from the rat hippocampal formation to the lateral and medial prefrontal cortex. Hippocampus 7: 397–402. 9287079 10.1002/(SICI)1098-1063(1997)7:4<397::AID-HIPO5>3.0.CO;2-G

[22] Van De Werd HJJM, Rajkowska G, Evers P, Uylings HBM (2010) Cytoarchtectonic and chemoarchitectonic characterization of the prefrontal cortical areas in the mouse. Brain Struct Funct 214: 339–353. 20221886 10.1007/s00429-010-0247-z PMC2862954

[23] Clarke JR, Cammarota M, Gruart A, Izquierdo I, Delgado-Garcia JM (2010) Plastic modifications induced by object recognition memory processing. Proc Natl Acad Sci U S A 107: 2652–2657. 20133798 10.1073/pnas.0915059107 PMC2823877

[24] Coutellier L, Würbel H (2009) Early environmental cues affect object recognition memory in adult female but not male C57BL/6 mice. Behav Brain Res 203: 312–315. 19427334 10.1016/j.bbr.2009.05.001

[25] Stanahan AM (2011) Similarities and differences in spatial learning and object recognition between young male C57Bl/6J mice and Sprague-Dawley rats. Behav Neurosci 125: 791–795. 21942439 10.1037/a0025133 PMC3187565

[26] Sannino S, Russo F, Torromino G, Pendolino V, Calabresi P, et al (2012) Role of the dorsal hippocampus in object memory load. Learn Mem 19: 211–218. 22523415 10.1101/lm.025213.111

[27] Ennaceur A, Aggleton JP (1997) The effects of neurotoxic lesions of the perirhinal cortex combined to fornix transection on object recognition memory in the rat. Behav Brain Res 88: 181–193. 9404627 10.1016/s0166-4328(97)02297-3

[28] Wan H, Aggleton JP, Brown MW (1999) Different contributions of the hippocampus and perirhinal cortex to recognition memory. J Neurosci 19: 1142–1148. 9920675 10.1523/JNEUROSCI.19-03-01142.1999 PMC6782155

[29] Hammond RS, Tull LE, Stackman RW (2004) On the delay-dependent involvement of the hippocampus in object recognition memory. Neurobiol Learn Mem 82: 26–34. 15183168 10.1016/j.nlm.2004.03.005

[30] Hannesson DK, Vacca G, Howland JG, Phillips AG (2004) Medial prefrontal cortex is involved in spatial temporal order memory but not spatial recognition memory in tests relying on spontaneous exploration in rats. Behav Brain Res 153: 273–285. 15219729 10.1016/j.bbr.2003.12.004

[31] Sutherland RJ, Sparks FT, Lehmann H (2010) Hippocampus and retrograde amnesia in the rat model: a modest proposal for the situation of systems consolidation. Neuropsychologia 48: 2357–2369. 20430043 10.1016/j.neuropsychologia.2010.04.015 PMC2900526

[32] Gabbott PL, Warner TA, Jays PR, Salway P, Busby SJ (2005) Prefrontal cortex in the rat: projections to subcortical autonomic, motor, and limbic centers. J Comp Neurol 492: 145–177. 16196030 10.1002/cne.20738

[33] Barker GR, Warbuton EC (2011) Evaluating the neural basis of temporal order memory for visual stimuli in the rat. Eur J Neurosci 33: 705–716. 21226775 10.1111/j.1460-9568.2010.07555.x

[34] Rosen GD, Harry JD (1990) Brain volume estimation from serial section measurements: a comparison of methodologies. J Neurosci Methods 35: 115–24. 2283883 10.1016/0165-0270(90)90101-k

[35] Mouton PR (2002) Principles and practices of unbiased stereology: an introduction for bioscientists. Baltimore: The John Hopkins University Press. 214 p.

